# Advances in the prevention and management of procedural bleeding in patients with cirrhosis

**DOI:** 10.1007/s12072-025-10834-2

**Published:** 2025-07-08

**Authors:** Jessica P. E. Davis, Alberto Q. Farias, Nicolas M. Intagliata

**Affiliations:** 1 Division of Gastroenterology and Hepatology, DC Veterans Affairs Medical Center, Washington, DC USA; 2 Department of Gastroenterology, University of Sao Paulo School of Medicine, Sao Paulo, Brazil; 3 Division of Gastroenterology and Hepatology, University of Virginia, Charlottesville, VA USA

**Keywords:** Thrombosis, Procedure, Bleeding, Hemostasis

## Abstract

**Introduction:**

Patients with cirrhosis frequently require procedures and are at risk of bleeding related to interventions. Procedural bleeding adversely impacts patients with cirrhosis and is associated with mortality. Assessment of bleeding risk in these patients is complex due to changes in hemostasis, portal hypertension, elevated thrombosis risk, and comorbid infection and renal disease. This clinical review will discuss current data regarding risk assessment, prevention, and management of procedural bleeding in patients with cirrhosis.

**Discussion:**

Risk of procedural bleeding in patients with cirrhosis involves patient-related and procedure-related factors. Conventional hemostasis parameters such as prothrombin time and platelet count are not predictive of bleeding in cirrhosis and may lead providers to overestimate bleeding risk. Hepatic decompensation, kidney injury, metabolic syndrome, alcohol use, and infections are all associated with increased bleeding risk. Procedure type, urgency, and operator experience also influence procedural bleeding risk. Historically pre-procedural transfusion support has been used in attempt to mitigate procedural bleeding risk. However, mounting data argues against this approach. Patient optimization, procedure conditions, and procedure technique can minimize bleeding risk. Viscoelastic testing may be useful to reduce the use of prophylactic transfusion and reassure proceduralists.

**Conclusion:**

Historically, the risk of procedural bleeding has been overestimated in patients with cirrhosis due to abnormal conventional coagulation testing including prolonged prothrombin time and thrombocytopenia. Prophylactic transfusion has not been consistently demonstrated to reduce bleeding risk and carries some risks. Performing only necessary procedures under optimal conditions with safe technique and preparation for rescue transfusion can minimize procedural-associated bleeding and its consequences.

## Introduction

Performing invasive procedures in patients with cirrhosis presents a unique set of challenges. Patients with chronic advanced liver disease are an inherently high-risk population prone to recurrent complications. They frequently require hospitalizations and procedures and have higher risk of mortality compared to patients without liver disease [1]. Historically, patients with cirrhosis have been thought to be at higher risk of both spontaneous and procedural bleeding. Estimation of procedural bleeding risk is not only a common clinical conundrum given the frequency of procedures, but also highly impactful as procedural bleeding is linked with increased mortality. In one large prospective study, hospitalized patients with cirrhosis who had bleeding associated with procedures had significantly higher 30-day mortality (aOR, 3.14; 95% CI, 1.66–5.97; *p* < 0.001) [1].

**Fig. 1:**
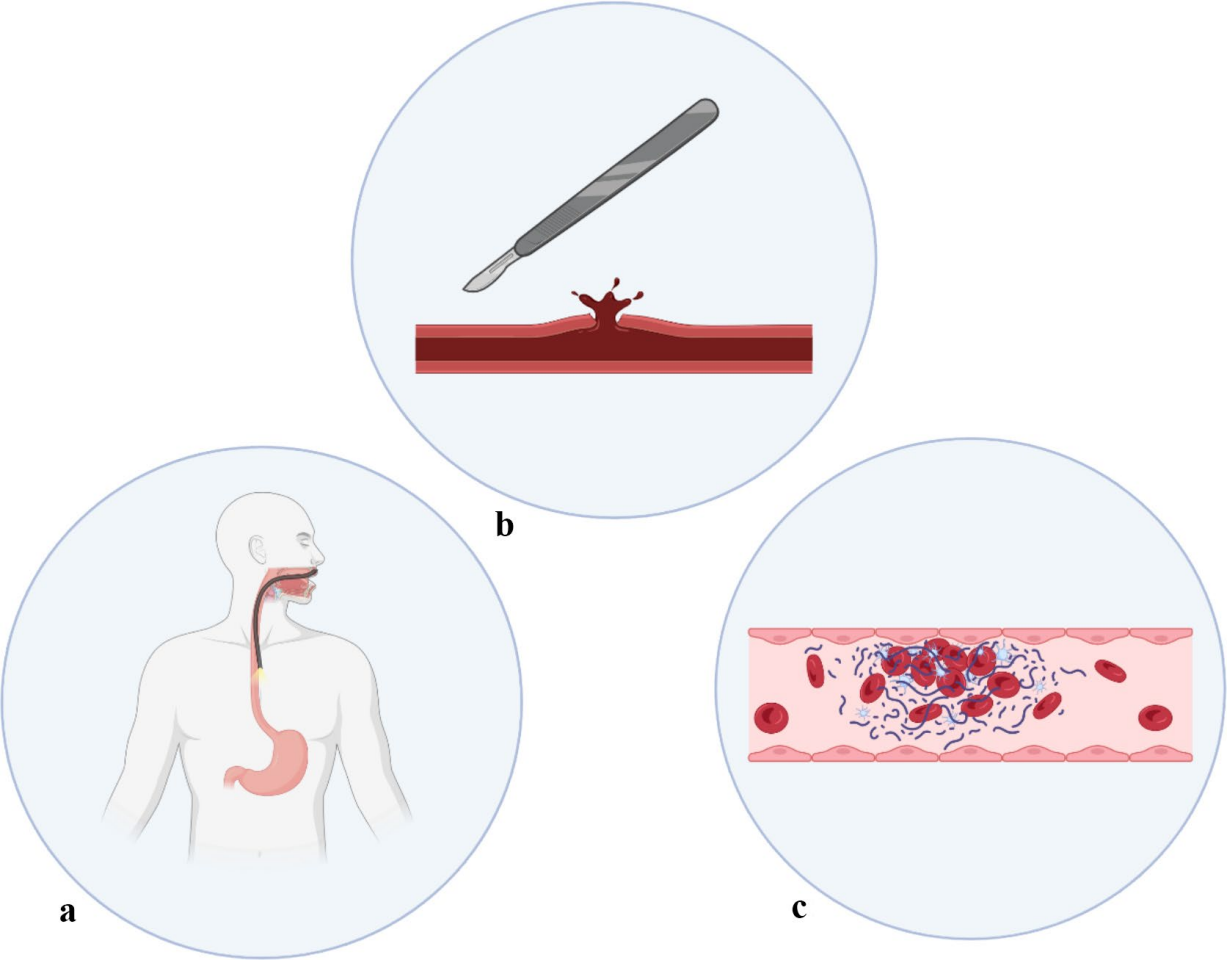
Types of procedural bleeding in patients with cirrhosis. **a** Portal hypertensive bleeding. **b** Mechanical vessel injury. **c** Hemostasis failure

Procedural bleeding in patients with cirrhosis can arise from multiple factors (Fig. 1), with portal hypertension being an important driver. Portal hypertension leads to the formation of large portosystemic collaterals, increasing the risk of spontaneous bleeding, such as esophageal variceal hemorrhage, and resulting in large collaterals that can pose significant hazards during abdominal and thoracic procedures.

When hospitalized, patients with cirrhosis may experience systemic complications including acute-on-chronic liver failure (ACLF), infection, and kidney injury. These conditions can potentially complicate the prediction of bleeding risk through alteration of the hemostatic system [2]. Infection, for example, is common in patients with cirrhosis with a prevalence of nearly 30% in patients with liver disease admitted with an acute decompensation and has been associated with release of endogenous heparinoids [2, 3]. Hemostasis is further altered in the setting of alcohol-related hepatitis, another common driver for acute decompensation in patients with liver disease [4].

Finally, managing anticoagulation in patients with cirrhosis before procedures raises additional concerns, as anticoagulation protocols are increasingly utilized in this population due to a growing recognition of the risk of venous thromboembolism (VTE) [5]. Considering the cardiac comorbidities associated with metabolic associated steatotic liver disease (MASLD), even more patients with liver disease may have indications for therapeutic anticoagulation (i.e., atrial fibrillation) in the future.

**Fig. 2:**
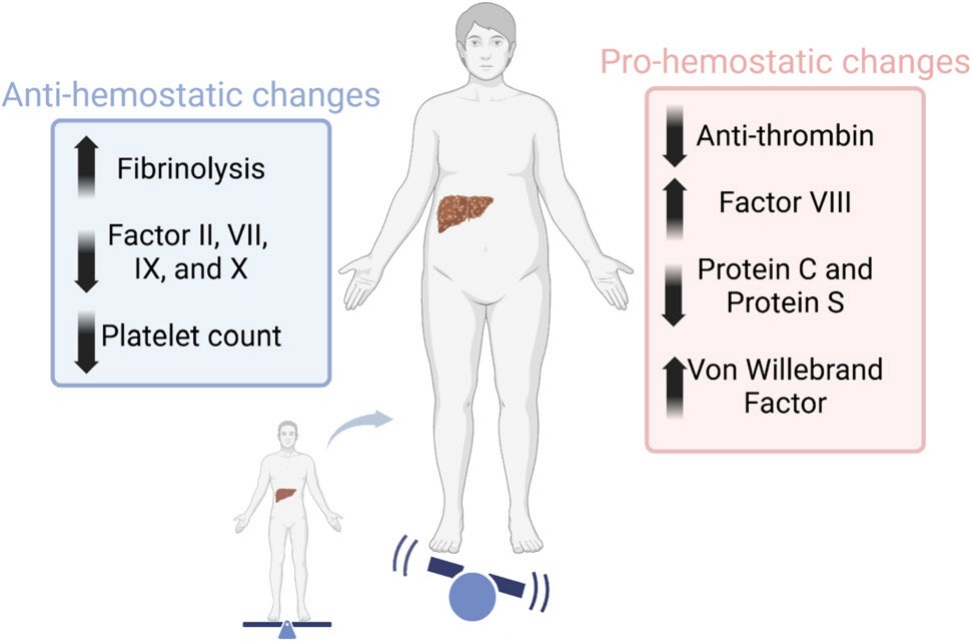
Rebalanced hemostasis in chronic liver disease

Cirrhosis is also associated with measurable changes to hemostasis ex vivo due to decreased hepatic synthetic function [6]. Clinically, patients with cirrhosis are at high risk of both bleeding and thromboembolism [7]. Some patients with hemostatic failure may have prolonged oozing from puncture sites or mucosa (e.g., oozing after dental extraction). Modern understanding is that hemostasis in liver disease is “rebalanced” with multiple changes impacting coagulation, not all of which are captured by conventional laboratory assessment (Fig. 2) [8]. For example, prolonged PT/INR and thrombocytopenia are common laboratory findings in patients with cirrhosis. Decreased procoagulant factor levels cause prolongation of INR but concomitant decreases in anticoagulant proteins that, while not captured in the INR, counterbalance these changes in vivo. Similarly, while thrombocytopenia is common in patients with cirrhosis, increased von Willebrand’s factor and factor VIII and decreased von Willebrand factor-cleaving protease AdamTS-13 serve to counterbalance the quantitative deficiency of platelets [6, 9]. There is evidence that thrombin generation is preserved even in patients with cirrhosis with platelets as low as 56 × 10^3^/µL [10]. While certainly relevant to bleeding risk in patients on vitamin K antagonists (VKA) or those with thrombocytopenia from other etiologies (e.g., hematologic processes), neither PT/INR nor platelet count is predictive of procedural bleeding risk in patients with cirrhosis [1, 11–23].

Providers who primarily care for patients with liver disease will likely appreciate the complexity of rebalanced hemostasis in cirrhosis and the limitations of conventional testing to predict procedural bleeding risk. However, proceduralists with a broader scope of practice may be less familiar with practice standards and more hesitant to abandon prophylactic transfusion in patients with abnormal coagulation parameters [24]. This further complicates care of these patients and underscores the need for multidisciplinary care in this high-risk group. Prophylactic transfusion to “correct” laboratory abnormalities has not been shown to reduce bleeding risk. In a prospective study [25], evidence of normal or high thrombin generation was found in 94% of patients before fresh frozen plasma transfusion, even in those with bacterial infections, which have been shown to impair coagulation through the release of endogenous heparinoids [26]. Transfusion before invasive procedures enhanced the total amount of generated thrombin by 5.7%, and less than 2% of patients had values revert to the normal range after transfusion. These responses were similar in patients with compensated or decompensated cirrhosis, ACLF, infection, or shock. Transfusion even appears to slightly decrease thrombin generation in about a third of patients, presumably by replenishing protein C.

In addition, transfusion is costly and carries real risks including risk of transfusion-related lung injury (TRALI), transfusion related circulatory overload (TACO) and transfusion-related immunomodulation (TRIM). Transfusion may ultimately increase portal pressure and increase the risk of portal-hypertension-related bleeding [27]. Furthermore, patients with cirrhosis are at high risk of venous thromboembolism (VTE) [28] and some hemostatic and antifibrinolytic agents have been linked with higher rates of VTE so this risk must be appreciated as well when using these products [29].

Data examining procedural bleeding in patients with cirrhosis are largely retrospective and observational with the notable exception of the large, prospective, multicenter PROC-BLEED trial [1]. Prospective, randomized trials examining the utility of interventions to reduce procedural bleeding are logistically challenging due to the low frequency of procedural bleeding in cirrhosis and the lack of control arms where patients with hemostatic changes do not receive prophylactic transfusion support [30]. Accepting this limitation, there are relevant lessons in available data that challenge current clinical practice.

This review will synthesize the current evidence on procedural related bleeding in patients with cirrhosis with a focus on prevention and management strategies. Data from varied procedure types including surgical, endoscopic, and common hospital-based procedures will be included in the context of pertinent societal guidelines [7, 30, 31].

## Risk assessment

**Fig. 3:**
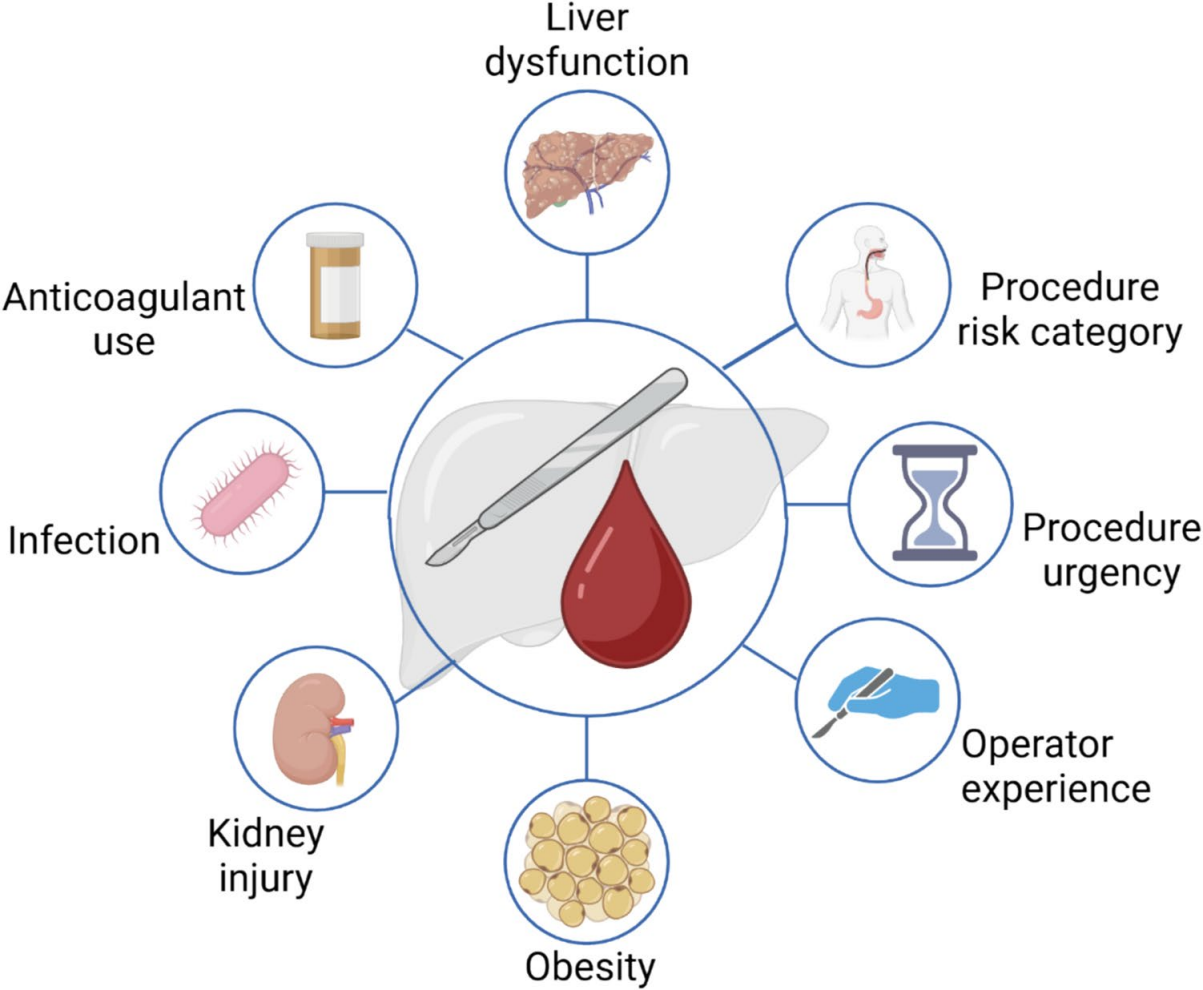
Risks associated with procedural bleeding in patients with cirrhosis

Pre-procedure risk assessment is critical to ensure safety for all patients undergoing invasive procedures but is particularly important in patients with cirrhosis. Patients with cirrhosis have high rates of hospitalization and frequently require procedures. It is useful to summarize risk assessment into two broad categories: patient-related risks and procedure-related risks. Several patient characteristics have been associated with increased risk of procedural bleeding in patients with cirrhosis (Fig. 3).

### Patient-related risk factors

#### Severity of liver disease

Severity of synthetic liver dysfunction is associated with risk of bleeding in multiple studies. Specifically, increases in both MELD score and CTP score are correlated with increase in bleeding events [1, 21]. In a prospective cohort study MELD > 25 was predictive of bleeding events for hospitalized patients with cirrhosis undergoing non-surgical procedures and the average MELD among patients who bled was 24.5 compared to 18.5 among patients who did not experience a bleed [1]. In the same study, 86% of patients who had a bleeding event were CTP class C versus 55% of patients who underwent procedures and did not bleed [1]. While INR is a component of the MELD score, elevated INR is not independently predictive of bleeding risk as detailed below.

In addition to chronic advanced liver disease, acute-on-chronic liver failure (ACLF) also increases risk of bleeding [32]. Patients with ACLF have been shown to have hypocoagulable changes in hemostasis with decreased thrombin generation [33] and slower clot initiation, prolonged clotting time, and decreased clot firmness on viscoelastic testing (VET) [34]. In a retrospective cohort study of post-paracentesis bleeding in ACLF patients, the rate of bleeding was high (2.99%) and low fibrinogen was predictive of increased bleeding risk in patients with ACLF undergoing procedures [35]. In one large prospective cohort study of hospitalized patients with cirrhosis undergoing non-surgical procedures, the relative risk of procedural bleeding was 2.05 (95% CI, 1.32–3.19) in patients with ACLF versus those without ACLF [1]. The association between bleeding events and ACLF was strongest at higher grade ACLF (grade II and grade III) [1].

Patients with portal hypertension are at higher risk of bleeding events versus those without portal hypertension in both surgical and bedside procedures. While many studies do not distinguish between types of bleeding, increased portal hypertension is associated with both increased risk of portal hypertensive bleeding and mechanical bleeding after interventions. This risk is explained in part due to increased hydrostatic pressure that leads to the development of large portosystemic collaterals that present hazards in the surgical or procedural field. Even in procedures without direct involvement of the splanchnic circulation, there is a higher risk of variceal hemorrhage in patients with more severe portal hypertension that has the potential to be acutely exacerbated by fluid resuscitation perioperatively. Finally, ascitic fluid has been demonstrated to have fibrinolytic effects and this should be considered for intraabdominal procedures in patients with ascites [36].

#### Hemostasis changes

Historically, patients with cirrhosis were thought to be at increased risk of bleeding due to coagulopathy evidenced by prolonged INR and thrombocytopenia. Modern understanding is that viewing these changes in INR and platelets in isolation is misleading as they represent only one aspect of a multitude of changes to hemostasis that occur in cirrhosis physiology. Concomitant decreases in pro-coagulant proteins and qualitative changes in platelet function in fact lead to fragile “rebalanced” hemostasis where both bleeding and clotting can occur [6] (Fig. 2).

While largely retrospective, many studies have examined the relationship between INR, platelet count, and procedural bleeding. Prothrombin time/INR and aPTT tests are terminated at endpoints corresponding to less than 5% of the reaction completion, meaning that 95% of the generated thrombin is not captured by these tests. Additionally, they cannot assess plasma anticoagulant activity because the reaction lacks thrombomodulin, the endothelial activator of protein C. Therefore, it is not surprising that INR fails to reflect procedural bleeding risk in cirrhosis [37].

Prolonged INR is not associated with increased risk of bleeding in the setting of surgical, endoscopic, or bedside procedures [7, 30, 31]. One large meta-analysis of more than 13,000 patients with cirrhosis showed no association between pre-procedure INR and bleeding events (pooled odds ratio 1.52; 95% CI 0.99, 2.33; *p* = 0.06) [15]. The same study showed no difference in mean INR in patients who had periprocedural bleeding versus those without bleeding.

Data on the relationship of platelet count and procedural bleeding are mixed and must be interpreted with caution as thrombocytopenia, in addition to its direct impact on primary hemostasis, can act as a proxy for more advanced portal hypertension. In one large prospective study of > 3000 non-surgical procedures in patients with cirrhosis, platelet count was not different among patients who experienced bleeding versus those who did not (118.8 k/μL vs 128.7 k/μL, *p* = 0.159) and was not predictive of bleeding (aOR 0.93 (0.65–1.27, *p* = 0.635) [1]. In another prospective study of 363 patients with cirrhosis undergoing 852 procedures, procedural bleeding was not associated with platelet count and in the ten patients with most profound hemostatic derangements (platelet count 3 k to 47 k) no procedural bleeding occurred [38]. Finally, one large meta-analysis that looked at a variety of procedures was not able to identify a consistent platelet cutoff that was predictive of procedural bleeding [39].

Several studies have evaluated the potential use of viscoelastic testing (VET) to predict procedural bleeding in cirrhosis. VET includes a whole blood sample, blood clot activator (e.g., kaolin) and mechanical force (Table 1). The amount of force and time it takes for a whole blood sample to form, sustain, and lyse a clot are reported. Widely used intra-operatively in cardiac disease and trauma surgery, VET is appealing in the setting of cirrhosis because, unlike many available assays, it evaluates whole blood and thereby may theoretically better capture nuances of hemostasis [40]. VET results are largely not impacted by severity of portal hypertension [41]. Disadvantages of VET include its limited availability in clinical practice and the lack of well-established norms for cirrhosis, which makes the interpretation of assay results highly dependent on significant expertise.

**Table 1:** Available viscoelastic testing modalities and platforms

Modality	Testing platform	Mechanical force	Quantification
Thromboelastography	TEG	Rotating cup containing suspended pin	Tracing wire affixed to the suspended pin
Rotational thromboelastometry	ROTEM	Stationary cup with a central rotating pin	Optical detection system
Sonorrheometry	Quantra	Ultrasound pulses	Ultrasound transducer

Use of VET in patients with cirrhosis decreases the use of blood products to correct INR and thrombocytopenia prior to invasive procedures across various contexts without associated increase in bleeding rates [42–45]. This evidence supports the utility of VET to reassure proceduralists and hepatology providers that a procedure may go forward without prophylactic transfusion; however, the use of VET as a predictor for procedural bleeding is not established. While smaller studies have shown a link between VET parameters and bleeding events [46, 47], in these three trials, there was not an association between VET findings and procedural bleeding.

#### Use of anticoagulation and antiplatelet agents

Several studies have shown that the use of prophylactic anticoagulation in patients with cirrhosis (e.g., VTE prophylaxis in hospitalized patients) is not associated with increased bleeding events in general [1]. In terms of common bedside procedures, in one large prospective observational study, use of any antithrombotic medications within 24 h of a procedure was not associated with procedural bleeding (27.8% of procedures without bleeding vs 31.2% of procedures with bleeding, *p* = 0.198) [1]. On analysis of individual agents, clopidogrel at time of procedure was more common in procedures with bleeding (3.3% vs 0.5%, *p* = 0.019). Importantly, use of VTE prophylaxis at hospital admission was not associated with procedural bleeding.

In endoscopy, small retrospective studies have shown no difference in bleeding after endoscopic variceal ligation in patients on and off anticoagulation [48–50]. While there is no theoretical basis for anticoagulation to increase the risk of spontaneous variceal bleeding which is driven by portal pressure, not hemostatic derangements, there are plausible concerns that bleeding related to mechanical injury during ligation or from post-banding ulcer (PBU) formation could be exacerbated by anticoagulant use. One retrospective study of patients with decompensated cirrhosis undergoing variceal ligation while on anticoagulation reported that 9% of patients had post-procedural bleeding, comparable to published rates in patients not on anticoagulation [50].

Current guidelines address the issue of therapeutic anticoagulation and variceal ligation largely in the context of initiating anticoagulation for portal vein thrombosis. The recommendations vary, reflecting the uncertainty of available data. EASL recommends deferring initiation of therapeutic anticoagulation until variceal prophylaxis has occurred. It is worth mentioning that patients who have never had previous variceal bleeding may be started on non-selective beta blocker therapy for primary prophylaxis without the need to delay anticoagulation after endoscopy. For secondary prophylaxis, monotherapy with pharmacological treatment has been shown to be insufficient, and performing endoscopic prophylaxis before initiating anticoagulation is a common practice pattern.

In contrast, AASLD recommends initiation of anticoagulation as soon as possible, noting the lack of firm evidence regarding increased risk in those on anticoagulation. Meanwhile, the Baveno VII portal hypertension consensus guidance suggests variceal ligation can be performed in patients on vitamin K antagonists [7, 30, 51] and the Austrian Billroth IV consensus guidance suggests anticoagulation does not need to be interrupted for variceal ligation, noting that given the lag between EVL and PBU formation, anticoagulation would typically already be resumed even if held for the endoscopy [52].

In the context of transplantation, there are minimal data available on the impact of antithrombotic use on operative bleeding risk during the procedure. VTE prophylaxis is recommended in hospitalized patients with cirrhosis [30] and widely used [53]. Available data support that bleeding risk is similar amongst DOACs compared to VKA or LMWH in the setting of transplant [54]. Expert guidance recommends that patients on warfarin receive vitamin K or PCC but not fresh frozen plasma prior to transplant [55]. Authors also advise that the specific antidotes (e.g., idarizumab or andexanet-alpha), if available, be used to reverse DOACs prior to urgent transplant.

#### Metabolic syndrome and obesity

Outside of liver disease, obesity has been associated with increased mortality and morbidity for surgical, endoscopic, and bedside procedures [56]. Obesity has also been associated with increased risk of procedural bleeding in patients with cirrhosis [1]. As data are observational, it is unknown whether the driver of obesity and procedural bleeding rates in liver disease is increased technical difficulty due to habitus changes, specific changes to hemostasis associated with metabolic syndrome that increase the likelihood of bleeding, or other factors. The mechanism underlying the increased bleeding risk in obese patients appears to be complex, as metabolic liver disease is associated with pro-coagulant changes to hemostasis and increased risk of VTE [57, 58]. This highlights the importance of avoiding unnecessary pro-thrombotic treatments which may enhance the baseline risk of VTE and minimizing unnecessary interruptions of prophylactic anticoagulation.

#### Kidney injury

A variety of hemostatic abnormalities has been observed in patients with acute kidney injury and chronic kidney disease [59]. The most common include prolonged bleeding time, defective platelet aggregation, and reduced platelet adhesiveness. The pathophysiology is not fully understood, but proposed mechanisms involve platelet inhibition by plasma metabolites such as urea, guanidinosuccinic acid, and phenolic acid. Additional factors include increased vessel wall prostacyclin, abnormal platelet arachidonic acid metabolism, defective binding of the factor VIII complex, impaired platelet-vessel wall interaction due to severe anemia, and platelet storage pool deficiency. However, VET in uremic subjects shows significantly increased amplitudes, which correlate positively with fibrinogen concentration—commonly elevated in chronic renal failure—and negatively with hematocrit levels. Paradoxically, thromboelastography indicates hypercoagulability, despite the prolonged bleeding time typically observed in uremic patients [60, 61]. Patients with AKI and cirrhosis have been shown to have changes to hemostasis on VET and decreased factor VIII levels that may contribute to bleeding risk [62, 63].

From a clinical perspective, both acute kidney injury (AKI) and chronic kidney disease (CKD) have been associated with post-procedural bleeding risk in patients with cirrhosis [2, 63]. Inpatients with post-procedural bleeding in a variety of bedside procedures were more likely to have both AKI (54.9% vs 33.6%, *p* < 0.001) and CKD (25.6% vs 17.3%, *p* = 0.011) [1]. In one retrospective study of 86 patients with cirrhosis who had bleeding post-paracentesis, AKI increased the risk of bleeding fourfold despite controlling for comorbid sepsis [64]. In another large retrospective cohort review of 265 patients who underwent variceal ligation, the mean creatinine among those who had post variceal bleeding was 2.2 mg/dL compared to a mean of 1.0 mg/dL among patients without bleeding (*p* = 0.001) [48].

#### Infection

Infection in patients with cirrhosis has been associated with increased endogenous heparin production, demonstrable in VET, and other changes in hemostasis that improve with resolution of infection [26, 65]. Infection at admission was more common in a cohort study of inpatients with cirrhosis undergoing nonsurgical procedures (44% vs 24%, *p* < 0.001) [1]. Antibiotic prophylaxis for gastrointestinal bleeding in patients with cirrhosis has been shown in a large meta-analysis to decrease the risk of re-bleeding (RR 0.53, 95% CI 0.38–0.74) and mortality (RR 0.79, 95% CI 0.63–0.98) [66].

### Procedure-related risk factors

#### Procedure type

Procedure characteristics also inform risk of procedural bleeding in patients with cirrhosis. Unfortunately, historically definitions used in literature evaluating procedural bleeding in cirrhosis have been variable, making comparison of studies difficult. There is growing consensus, however, to use standardized definitions based on expert guidance which stratify procedures into two categories, high risk and low risk (Table 2). Procedures at high risk of bleeding include those where risk of major bleeding is > 1.5%, difficult to control if occurs, or causes serious morbidity (e.g., central nervous system bleeding) [67]. Procedures at low risk of bleeding have rate of major bleeding < 1.5% and bleeding can be controlled if it occurs. Common high-risk procedures in patients with cirrhosis include biliary sphincterotomy, transjugular intrahepatic portosystemic shunt, endoscopy polypectomy, and dental extractions. Common low-risk procedures include paracentesis, central venous catheter placement, and diagnostic endoscopy and colonoscopy including biopsy. Of note, there is disagreement among both experts and societal guidelines for some procedures given the lack of available literature. In one recent survey of expert authors on bleeding risk from invasive procedures in cirrhosis, no consensus was achieved for bleeding risk stratification for 28 of the 80 procedures [68]. Endoscopic variceal ligation, for example, is considered low risk by AASLD and high risk by EASL [7, 30, 68].

**Table 2:** High- and low-risk procedures per EASL and AASLD guidelines (Northup 2020, EASL 2022)

	High risk	No consensus^a^	Low risk
Gastrointestinal	Endoscopic polypectomy; Endoscopic dilation; Endoscopic mucosal resection; Balloon-assisted enteroscopy; EUS with FNA; Cystgastrostomy	Endoscopic variceal ligation; ERCP; Percutaneous liver biopsy	Paracentesis; Diagnostic esophagogastroduodenoscopy; Enteroscopy; Colonoscopy with biopsy; Capsule endoscopy; EUS without FNA
Vascular	Transjugular intrahepatic portosystemic shunt; Transhepatic arterial chemo- or radio-embolization	Transjugular liver biopsy; Percutaneous liver cancer ablation	Central venous catheter insertion or removal; IVC filter placement; Diagnostic venography
Cardiothoracic	Interventional venography/angiography; Percutaneous thoracic organ biopsy; Bronchoscopy with biopsy		Diagnostic left and right heart catheterization; Transesophageal echocardiography; Diagnostic bronchoscopy; Thoracentesis
Dental	Dental extraction		Dental cleaning/nonextraction procedures
Central Nervous System	Central nervous system procedures		
Other	Nephrostomy tube placement; Percutaneous nonliver abdominal solid organ biopsy; Intra-articular injections; Intra-ocular procedures		Skin biopsy

High-risk procedures are defined as those with > 1.5% chance of bleeding or those with significant morbidity/mortality should bleeding occurs (e.g., central nervous system bleeding)

^a^Procedure category by society for procedures with no consensus:

ERCP: High-risk per EASL, per AASLD only high risk if biliary sphincterotomy performed

Endoscopic variceal ligation: high-risk per EASL, low risk if “routine” per AASLD

Percutaneous liver biopsy: low-risk per EASL, high-risk per AASLD

Transjugular liver biopsy: low-risk per EASL, high-risk per AASLD

Percutaneous liver cancer ablation: low-risk per EASL, high-risk per AASLD

#### Operator experience

Intuitively, operator inexperience may increase the risk of procedural bleeding; however, this is not firmly established in available literature. Trainee participation was not associated with risk of bleeding in a range of bedside procedures in a recent prospective study [1]. In paracenteses, for example, providers with as few as 10 supervised procedures had low complications when they performed subsequent paracenteses independently [13]. Finally, in multiple procedures in patients with cirrhosis, inexperienced operators have been associated with less procedural bleeding, underscoring the potential for selection bias in these retrospective studies [21, 69].

#### Procedure urgency

Procedure urgency also influences risk of bleeding with emergent procedures at a higher risk of bleeding than elective procedures. Emergent procedures are more likely to occur in patients in high-risk characteristics (e.g., ACLF, sepsis, AKI). Furthermore, proceduralist teams have less ideal operating conditions in emergent settings with potential changes in procedure setting, supplies, and staffing. It is well-established that overall mortality is significantly higher in patients with cirrhosis who undergo emergent vs elective procedures [70]. In terms of bleeding, in one retrospective study of patients undergoing variceal ligation, risk of bleeding was fourteen times higher in setting of emergent endoscopy versus elective cases (7.1% vs 0.5%, *p* < 0.001) [71].

## Prevention strategies

### Pre-procedural risk assessment

Careful consideration of the risk factors outlined above can inform the risk of bleeding prior to procedures. Analysis of both patient- and procedure-related characteristics gives the most holistic assessment. Procedural bleeding risk assessment is reviewed in multiple available societal guidelines as well [7, 30]. Both EASL and AASLD guidelines emphasize delineating the bleeding risk of a particular procedure and avoidance of strategies that carry harm without proven benefit (e.g., prophylactic transfusion). A particular focus on which risk factors are static versus modifiable aids in optimization of these high-risk patients. Delays in procedures that are not emergent for patients with infection or kidney injury to allow these comorbidities to improve, for example, could potentially reduce the risk of bleeding.

There are no standard laboratory tests that predict procedural bleeding in patients with cirrhosis and current guidelines do not recommend obtaining coagulation testing including PT/INR, platelet count, fibrinogen [30]. VET can be useful as baseline to guide resuscitation in the event bleeding occurs, particularly in procedures that are at high risk, emergent, or where local hemostasis is not possible [30].

### Pre-procedural optimization

#### Modifiable patient risk factors

After risk assessment, focusing on modifiable risk factors is recommended to minimize bleeding risk. In this high-risk cohort, the initial key question is whether an invasive procedure can be avoided, or, in the acutely ill patient, delayed, to allow improvement in modifiable risk factors. For example, a short delay to allow improvement in kidney function or infection control. In addition, providers could consider longer delays giving time for physical therapy for the frail patient or weight loss for patients with obesity who need more elective procedures. Portal hypertension should also be optimized as much as possible to ensure ideal operating conditions. Patients with ascites should be controlled with diuretics where possible or have paracenteses pre-procedurally, those with esophageal varices should be risk-reduced with beta blockade or ligation as indicated.

#### Surgical technique

Surgical technique can also be important in pre-procedure risk minimization. In patients with known abdominal portosystemic collateral vessels who need abdominal surgery, participation of a liver transplant surgeon who has experience operating in the setting of portal hypertension is ideal. Imaging guidance where possible to avoid vascular injury and confirm anatomical landmarks can also reduce bleeding risk. Use of ultrasound guidance, for example, has been shown to decrease the risk of bleeding in the setting of thoracentesis and decrease risk of capsule perforation TIPS placement in patients with cirrhosis [72, 73].

#### Pre-operative TIPS

Given that much of the increased peri-operative morbidity and mortality in patients with cirrhosis appears to be driven by portal hypertension, there has been longstanding interest in the potential for pre-surgical TIPS placement to improve perioperative outcomes in patients with cirrhosis and CSPH. Several small series have examined the relationship between TIPS placement and outcomes and the impact remains uncertain. One recent propensity-matched study of 210 individuals compared those who had TIPS within six months of an elective surgery to those who did not [74]. The pre-surgical TIPS cohort had an increased mortality and no difference in intra-operative blood loss [74]. Given the uncertainty of available data, TIPS placement prior to elective surgery is not recommended outside of standard indications.

#### Prophylactic transfusion of blood products

In patients with measurable changes to hemostasis, several studies have evaluated the role of prophylactic transfusion. Despite the lack of proven efficacy, pre-procedure transfusions are commonly administered in clinical practice to patients with prolonged PT/INR or thrombocytopenia. However, there is no evidence to support that these transfusions reduce bleeding risk; instead, it has been shown that transfusions carry risks and increase costs. Ideally, a study examining the relationship between prophylactic transfusion and procedural bleeding risk would blindly randomize patients with coagulopathy to a transfusion arm versus no transfusion arm where only rescue transfusion was permitted. The scale required to adequately power such a trial is daunting taking into consideration the low frequency of bleeding in patients with cirrhosis undergoing procedures [30]. In fact, the only published study to date that has examined this focused on the impact of transfusions on bleeding after central venous catheter (CVC) placement in patients with cirrhosis. However, the study was not adequately powered, with only 57 subjects enrolled [65].

Acknowledging this limitation, there are observational data available to guide practice. In the prospective PROC-BLeeD study, procedures with prophylactic transfusions had higher rates of bleeding than those without prophylactic transfusion (19.4% vs 7.4%, *p* = 0.001) [1]. This was true for both pre-procedure platelet transfusion and pre-procedure plasma transfusions. Among patients with platelet count below 50,000, 28.6% of patients who had bleeding received pre-procedure platelet transfusion vs 10.4% of with thrombocytopenia without bleeding (*p* = 0.55) [1]. Similarly, among patients with INR > 1.5 who had procedural bleeding, 14% received plasma pre-procedure versus 3.5% of patients with INR > 1.5 without procedural bleeding (*p* = 0.001) [1]. In a randomized controlled trial that used viscoelastic testing to guide transfusions prior to a range of procedures in patients with cirrhosis, patients in the VET arm received significantly less transfusions and had no higher rates of bleeding [42]. Finally, while observational data has shown hypofibrinogenemia to be associated with bleeding in patients with cirrhosis [75], it is unclear if low fibrinogen levels represent a causal factor in bleeding or are a marker of critical illness and correction of fibrinogen levels with cryoprecipitate has not been consistently demonstrated to impact bleeding or mortality [76].

There has also been interest in transfusion of whole blood or packed red blood cells (pRBC) to correct anemia pre-procedurally as anemia impairs platelet function by affecting platelet flow and activation [30]. Limited observational data has linked anemia to increased procedural bleeding risk in cirrhosis [16, 77] however there are not data to evaluate the impact of pre-procedure pRBC transfusion on procedural bleeding rates. Transfusion of pRBC carries risk of volume overload and subsequent worsening of portal hypertension, with restrictive transfusion strategies (goal hemoglobin 7–9 g/dL) associated with improved survival compared to liberal transfusion in the setting of gastrointestinal bleeding [78].

Reflecting the available literature, recent guidelines recommend against routine pre-procedural transfusion to correct prolonged INR, thrombocytopenia or hypofibrinogenemia [7, 30, 31] (Table 3). EASL guidelines note that in patients with very low platelet counts undergoing procedures at high risk of bleeding where local hemostasis is not possible, transfusion of platelets can be considered on a case-by-case basis [30].

**Table 3:** Selected societal guideline recommendations regarding prophylactic measures to reduce procedural bleeding risk

Societal guideline	Correction of thrombocytopenia	Correction of prolonged PT/INR	Correction of hypofibrinogenemia
AASLD 2020	No routine pre-procedure correction	No routine pre-procedure correction	No routine pre-procedure correction
	Recommends against TPO-R use		
EASL 2022	Consider platelets and/or TPO-R on case-by-case basis if plt < 50 k and high-risk procedure, and local hemostasis not possible	No routine pre-procedure correction	No routine pre-procedure correction
AGA 2021	Recommends against routine use of prophylactic blood products in those undergoing GI procedures other than those with “severe” derangements should have risk–benefit discussion	Recommends against routine use of prophylactic blood products in those undergoing GI procedures other than those with “severe” derangements should have risk–benefit discussion	Recommends against routine use of prophylactic blood products in those undergoing GI procedures other than those with “severe” derangements should have risk–benefit discussion
	Recommends against use of TPO-R agonists in low-risk procedures		

AASLD, American Association for the Study of Liver Diseases; EASL, European Association for the Study of the Liver; AGA, American Gastroenterology Association

#### Thrombopoietin receptor agonists

There has also been interest in the use of thrombopoietin receptor (TPO-R) agonists to reduce the risk of procedural bleeding in patients with cirrhosis and thrombocytopenia. Available agents approved by the US Food and Drug Administration and the European Medicines Agency include romiplostim, eltrombopag, avatrombopag, and lusutrombopag. However, only the latter two have been approved for the management of thrombocytopenia as an alternative to platelet transfusion in adult patients with chronic liver disease who are scheduled to undergo invasive procedure. They must be administered several days prior to a procedure so are not feasible for urgent procedures. TPO-R agonists have been shown to improve platelet count and reduce pre-procedure platelet transfusion [79–82]. While a recent meta-analysis [83] suggested these agents may reduce procedural-related bleeding, these results must be interpreted with caution as they include *pre*-procedure bleeding and bleeding of questionable clinical relevance. Furthermore, none of the original RCTs included in the meta-analysis demonstrated a difference in procedural bleeding [79–82]. AASLD guidelines recommend against the routine use of TPO-R agonists to prevent procedural bleeding and EASL recommends consideration on a case-by-case basis in patients with platelet count < 50 k undergoing procedures at high risk of bleeding where local hemostasis is not possible [7, 30].

#### Prophylactic use of factor concentrates

In view of the lack of data supporting the use of blood products to improve procedural risk as well as clinical concerns about risks associated with transfusion including volume overload, there has been interest in use of hemostasis-promoting agents and concentrated products.

Prothrombin complex concentrates (PCC) are pooled products containing combinations of multiple vitamin-K dependent procoagulant factors, protein C, and protein S. Designed to treat supratherapeutic INR related to vitamin K antagonist use, PCC are appealing in cirrhosis due to their relatively low volume. While PCC are effective at reducing INR in cirrhosis [84], the clinical relevance of this is uncertain as INR does not reflect bleeding risk in cirrhosis. There are some pharmacokinetic data suggesting PCC are potentially more thrombotic in patients with cirrhosis than those without cirrhosis leaving dosage uncertain [84]. Use of PCC reduces blood product use in liver transplantation, but these data are limited to small retrospective observational studies [85, 86]. PCC use has been associated with thrombosis in the setting of cirrhosis [87]. The use of PCC to reduce the risk of procedural bleeding is not supported by societal guidelines [7, 30]. Similarly, concentrated recombinant factor VIIa was not shown to reduce bleeding in the setting of variceal hemorrhage and is not routinely recommended [88].

#### Prophylactic use of antifibrinolytic agents

Some patients with cirrhosis have evidence of hyperfibrinolysis both in vitro and clinically with oozing at mucosal wounds and puncture sites. There are antifibrinolytic agents available; previously aprotinin, which is now discontinued for concern for increased mortality in the context of cardiac procedures and now tranexamic acid (TXA). TXA is available in topical, oral, and intravenous forms. While aprotinin has some data showing reduction in use of blood products when used in liver transplant [89], there are not comparable data for TXA in this context [90]. TXA did not reduce bleeding or mortality in patients with gastrointestinal bleeding in a blinded RCT [29]. Of note, patients with cirrhosis in that study who received TXA had higher rates of thrombosis than those who did not. TXA has also been used topically for bleeding after dental procedures. A meta-analysis supported use of topical TXA in patients on therapeutic anticoagulation undergoing dental extractions [91] but no benefit was seen in a small prospective trial of liver transplant candidates undergoing dental extractions [19]. Routine use of antifibrinolytic agents to prevent procedural bleeding is not recommended by societal guidelines [7, 30].

## Intra-procedural strategies

### Classification of bleeding events

The definition of procedural bleeding is varied in available cirrhosis literature. The literature would benefit from standardization of bleeding definitions to improve generalizability and ability to assess the efficacy of interventions. The International Society of Thrombosis and Haemostasis has definitions for major bleeding and clinically relevant non-major bleeding in the setting of procedures (Table 4) [67, 92]. Major bleeding events are those related to procedures with hemoglobin drop of 2 g/dL or requiring transfusion of ≥ 2 units of pRBCs or that have a substantial clinical outcome (e.g., death, central nervous system bleed, reoperation required). Clinically relevant non-major bleeding (CRNMB) events are those that do not meet major bleeding criteria but require face-to-face evaluation or increase in level of care and minor bleeds are those that do not meet criteria for major bleeding or CRNMB.

**Table 4:** International Society of Thrombosis and Haemostasis definitions for major bleeding and clinically relevant non-major bleeding in the setting of procedures (Schulman 2010, Katz 2015)

Bleeding type	Bleeding characteristics
Major bleeding	Fatal bleeding and/or
	Bleeding that is symptomatic and occurs in a critical area or organ, such as intracranial, intraspinal, intraocular, retroperitoneal, pericardial, in a non‐operated joint, or intramuscular with compartment syndrome, assessed in consultation with the surgeon, and/or
	Extrasurgical site bleeding causing a fall in hemoglobin level of ≥ 2 g/dL or leading to transfusion of ≥ 2 units of whole blood or red cells within 24–48 h of bleeding and/or
	Surgical site bleeding that requires a second intervention or interferes with rehabilitation by delaying mobilization or delayed wound healing, resulting in prolonged hospitalization or a deep wound infection, and/or
	Surgical site bleeding that is unexpected and prolonged and/or sufficiently large to cause hemodynamic instability, as assessed by the surgeon. There should be an associated fall in hemoglobin level of ≥ 2 g/dL or leading to transfusion of ≥ 2 units of whole blood or red cells within 24 h of bleeding
Clinically relevant non-major bleeding (CRNMB)	Any sign or symptom of hemorrhage (e.g., more bleeding than would be expected for a clinical circumstance, including bleeding found by imaging alone) that does not fit the criteria for the ISTH definition of major bleeding but does meet at least one of the following criteria: requiring medical intervention by a healthcare professional; leading to hospitalization or increased level of care; prompting a face-to-face evaluation
Minor bleeding	Bleeding that does not meet criteria for major bleeding or CRNMB

CRNMB, clinically relevant non-major bleeding; ISTH, International Society of Thrombosis and Haemostasis

### Anesthetic and surgical considerations

Given the perioperative morbidity and mortality associated with cirrhosis, particularly those with decompensated cirrhosis or ACLF, this group requires particular attention in terms of sedation and surgical technique. Avoiding overtransfusion and volume overload has been associated with improved survival and reduced bleeding [78], potentially due to avoiding exacerbation of portal hypertension. Similarly, avoiding targeting excessively high mean arterial procedures for fluid resuscitation may help maintain portal pressures, with maintenance of a low central venous pressure being shown to reduce blood loss during transplant and hepatectomy [93, 94]. Maintenance of normothermia and avoiding metabolic acidosis are beneficial in this group to optimize hemostasis as well [95].

In patients with portal hypertension, planning for participation of a surgeon with experience operating in this group (i.e., a transplant surgeon), anticipating prolonged operating times and ensuring meticulous technique can potentially reduce the risk of bleeding. In liver transplantation, for example, use of modern techniques such as the piggyback approach (preservation of recipient inferior vena cava (IVC) to avoid total IVC clamping and maintain venous return) have resulted in less intraoperative blood loss [95]. Intraoperative blood cell salvage and autotransfusion using a cell saver machine can be employed during liver transplants or resections to reduce the need for allogeneic blood transfusions. Finally, patients benefit from receiving care at tertiary hospitals with significant experience in patients with advanced liver disease and multi-disciplinary teams.

### Intra-procedure hemostasis monitoring

Intra-operative monitoring of hemostasis, particularly in those patients with procedural bleeding, can be useful to guide transfusion support. VET can be useful intra-operatively for transfusion support, offering the benefit of rapid turnaround and whole blood analysis. VET have been shown to decrease the use of blood products intra-operatively in the setting of cirrhosis in multiple contexts including non-variceal gastrointestinal bleeding and liver transplantation [43, 45]. In both randomized trials, patients who were enrolled in the VET-guided transfusion arm received less blood products overall without higher rates of uncontrolled or recurrent bleeding.

## Post-procedural rescue strategies

### Post-procedural monitoring

Patients with cirrhosis warrant close monitoring for post-procedural bleeding. Patients, caretakers, and bedside providers should all receive education on signs and symptoms of bleeding. When being discharged from a monitored setting, caretakers and patients should receive an action plan for if acute bleeding occurs as well as reasons to return for urgent evaluation. Patients with risk factors for bleeding, e.g., high-risk procedure, decompensated, elevated MELD score, ACLF, may require prolonged post-operative observation and, particularly if there is potential for occult bleeding, consideration of serial hemoglobin monitoring.

### Bleeding post-variceal ligation

Esophageal variceal ligation (EVL), particularly in the setting of acute variceal bleeding, may result in procedural bleeding most commonly for two reasons, rebleeding of varices or post-banding ulcer formation. Patients with rebleeding varices after endoscopic intervention should be evaluated for emergent transjugular intrahepatic portosystemic shunt (TIPS) placement, as rebleeding varices are associated with high morbidity and mortality. Furthermore, consideration of “pre-emptive” TIPS is now recommended in a population at high risk of rebleeding—those with CTP C10-C13 or CTP B7-B9 with active bleeding at endoscopy—as this reduces rebleeding and improves survival [96]. Management of PBU bleeding is challenging and non-standard. PBU bleeding occurs in approximately 5% of EVL cases overall and is slightly more common after urgent EVL [97]. Proton pump inhibitors and sucralfate have been used for PBU bleeding but do not have established efficacy [97]. Vasoactive medications to address portal hypertension can also be used and endoscopic technique is variable including re-banding, sclerotherapy and, in refractory cases, balloon tamponade or esophageal stenting may be used [97]. While not widely reported for PBU specifically, hemostatic powders are a novel endoscopic tool that have been used successfully for treatment of PBU [98]. TIPS has also been used effectively in some cases of severe PBU bleeding [97].

## Emerging therapies and future directions

Assessing procedural bleeding risk in cirrhosis is challenging, as it involves a range of procedures and settings, the potential for multiple procedures in a single hospitalized patient, and the intricate nature of advanced liver disease management, particularly in patients with decompensated liver disease, high MELD, or ACLF.

Ongoing research is needed. In particular, a trial randomizing individuals with cirrhosis to no prophylactic transfusion versus an intervention arm to properly assess impact of interventions on bleeding is needed. Such a study would require substantial funding and societal backing, as the necessary scale demand each arm to include over 1000 patients, reflecting the low rates of procedural bleeding in cirrhosis [30].

A trial focusing on high-risk patients undergoing high-risk procedures would be most practical, as it would target higher expected bleeding rates and address the area of greatest uncertainty, making it an ideal approach. While restricting the procedure type would limit generalizability, as even ‘high-risk’ procedures in patients with cirrhosis have relatively low overall bleeding rates, a trial demonstrating this could provide reassurance and help bolster practitioners' confidence in avoiding prophylactic transfusions for low-risk procedures as well.

There is a pressing need for more personalized medicine, utilizing precise coagulation profiling to better understand individual differences in hemostasis among patients with cirrhosis, which could help predict bleeding risks.

VET are the most promising currently used assays but are not widely available and have not been established as risk predictors in multiple studies. Finally, the role of machine learning and artificial intelligence in procedural bleeding has been explored but not yet applied to cirrhosis patients and ought to be evaluated [99].

## Conclusion

Overall risk of bleeding in cirrhosis is lower than historically perceived. Nevertheless, it crucial to better understand this risk, considering the high frequency of procedures required in patients with cirrhosis and the association between procedural bleeding and increased mortality.

Predicting and stratifying bleeding risk in patients with cirrhosis undergoing invasive procedures is challenging due to the complexity of this medical condition, which requires individualized evaluation and coordinated multidisciplinary care.

The severity of liver disease, presence of portal hypertension, ACLF, renal injury, obesity, and infection are patient-related factors that influence bleeding risk. Procedure-side factors that impact bleeding risk include procedure type, level of risk inherent to the procedure, and procedure urgency. Importantly, conventional testing such as PT/INR and platelet count do not predict procedural bleeding. Transfusion carries risk including TACO, TRALI and TRIM and pre-procedure transfusion in most circumstances should be avoided. While widely used historically, prophylactic transfusion of blood products does not reduce risk of procedural bleeding and is not supported by current societal guidelines. Focusing on avoiding or ideally timing invasive procedures may improve bleeding risk. VET, while not established to predict bleeding, can be useful to avoid over-transfusion without increasing bleeding rates. Large-scale, collaborative research is needed to improve personalization of bleeding risk stratification.

## Abbreviations

AASLD: American Association for the Study of Liver Disease
ACLF: Acute-on-chronic liver failure
ALF: Acute liver failure
aOR: Adjusted odds ratio
CI: Confidence interval
CTP: Child–Turcotte–Pugh
CVC: Central venous catheter
DOAC: Direct oral anticoagulant
EASL: European Association for the Study of the Liver
EVL: Endoscopic variceal ligation
INR: International normalized ratio
IVC: Inferior vena cava
MASLD: Metabolic-associated steatotic liver disease
MELD: Model for end-stage liver disease
PCC: Prothrombin complex concentrate
pRBC: Packed red blood cells
PT: Prothrombin time
PTT: Partial thromboplastin time
PBU: Post-banding ulcer
PVT: Portal vein thrombosis
TACO: Transfusion-associated circulatory overload
TIPS: Transjugular intrahepatic portosystemic shunting
TPO-R: Thrombopoietin receptor agonists
TRALI: Transfusion-related acute lung injury
TRIM: Transfusion-related immune modulation
TXA: Tranexamic acid
VET: Viscoelastic testing
VKA: Vitamin K antagonists
VTE: Venous thromboembolism
